# Triglyceride‐Glucose Index‐Based Nomogram for Predicting Short‐Term Mortality in Sepsis Patients

**DOI:** 10.1002/kjm2.70199

**Published:** 2026-04-02

**Authors:** Jing Zhang, Yu‐Jing Jiang, Ya‐Ting Lv, Tong‐Qin Li, Li Ma

**Affiliations:** ^1^ Department of Critical Care Medicine The Second Hospital & Clinical Medical School, Lanzhou University Lanzhou Gansu China

**Keywords:** insulin resistance, nomogram, sepsis, triglyceride‐glucose index

## Abstract

The triglyceride‐glucose (TyG) index, a marker of insulin resistance, is associated with outcomes in critical illness; however, its predictive role for 28‐day mortality in Asian patients with sepsis has not been established. To address this, the current investigation was designed to evaluate its prognostic significance and to construct a risk prediction model for short‐term mortality. This retrospective study analyzed sepsis patients admitted to the intensive care unit (ICU) of the Second Hospital of Lanzhou University from January 1, 2018 to December 31, 2023. Participants were randomly split into training/validation cohorts (7:3 ratio). Multivariate logistic regression identified independent predictors. A nomogram was constructed by these predictors and it was validated using ROC curves (ROC), calibration plots, and decision curve analysis (DCA). A nomogram integrating seven variables—age, respiratory failure within 48 h, multiple organ dysfunction syndrome (MODS) within 48 h, international normalized ratio (INR), lactate level, prognostic nutrition index (PNI), and TyG index—predicted 28‐day mortality with AUCs of 0.855 (training, 95% CI: 0.818–0.891) and 0.826 (validation, 95% CI: 0.764–0.887), outperforming SOFA and APACHE II scores. Calibration curves confirmed alignment with actual outcomes, and DCA demonstrated broad clinical utility. The TyG index is an independent predictor of 28‐day sepsis mortality. The validated nomogram based on the TyG index enhances risk stratification, aiding clinicians in early decision‐making to improve patient outcomes and care quality.

**Abbreviations**
ALB: albumin
ALT: creatinine; alanine transaminase
AST: aspartate aminotransferase
AUC: area under the curve
CRP: C‐reactive protein
DB: direct bilirubin
DCA: decision curve analysis
Hb: hemoglobin
HCT: hematocrit
HDL: high‐density lipoprotein
ICU: intensive care unit
INR: international normalized ratio
IR: insulin resistance
LDL: low‐density lipoprotein
MODS: multiple organ dysfunction syndrome
NE %: neutrophil ratio
NLR: neutrophil‐to‐lymphocyte ratio
PCT: procalcitonin
PLT: platelet
PNI: prognostic nutrition index
RDW‐CV: Coefficient of variation of red blood cell distribution width
RDW‐SD: Standard deviation of red blood cell distribution width
ROC: operating characteristic curve
TB: uric acid; urea nitrogen; total bilirubin
TC: total cholesterol
TG: fasting glucose; fasting triglycerides
TyG: triglyceride‐glucose
WBC: white blood cell

## 1 Introduction

Sepsis is a life‐threatening condition characterized by organ dysfunction resulting from a dysregulated host immune response to infection [1]. Its mortality and disability rates remain high, posing a major and persistent global health threat [2]. Among Chinese patients, mortality rates are particularly elevated (ICU incidence: 20%; 90‐day mortality: 35.5%) [3, 4]. Therefore, accurate prognosis prediction is crucial for enhancing survival rates, guiding treatment decisions, and optimizing resource allocation.

Most current prognostic models for sepsis primarily rely on SOFA or APACHE II scores [5], often overlooking indicators of glucose metabolism disruption caused by early stress. The triglyceride‐glucose (TyG) index, recognized as a reliable tool for assessing insulin resistance [6], has been widely applied in evaluating metabolic abnormalities [7], ischemic stroke [8], among other conditions. Studies indicate that an elevated TyG index is significantly associated with an increased risk of in‐hospital mortality in critically ill sepsis patients [9]. There are differences in the clinical manifestations and risk factors of metabolic diseases between Asian and Western populations. The high‐carbohydrate dietary habits prevalent in Asia may enhance the prognostic value of the TyG index for predicting short‐term outcomes in sepsis. However, the application of this index in Asian populations remains under‐investigated. Therefore, validating the prognostic significance of the TyG index in Asian sepsis patients is of considerable importance.

This study aimed to develop and internally validate a TyG index‐based nomogram for predicting short‐term mortality in sepsis patients from a Western China cohort.

## 2 Methods

### 2.1 Patients

Patients with sepsis admitted to three comprehensive ICUs at Lanzhou University Second Hospital from January 1, 2018 to December 31, 2023 were enrolled. The enrolled cohort was predominantly of Han ethnicity (524 patients, 90.6%), followed by Hui and Dongxiang ethnicities (42 patients, 7.3%), with a small number of patients from other ethnic groups including Tibetan (12 patients, 2.1%). Inclusion criteria:(1) Diagnosis of sepsis according to Sepsis‐3 criteria; (2) Age ≥ 18 years; (3) ICU stay ≥ 24 h. Exclusion criteria: (1) Pregnant or lactating women; (2) Without fasting glucose or triglyceride data, loss to follow‐up, or abandonment of treatment; (3) Use of corticosteroids prior to admission; (4) diabetes, autoimmune diseases, or end‐stage malignancy; (5) End‐stage renal failure (Figure S1). The study adhered to the principles of the Declaration of Helsinki and was approved by the ethics committee of Lanzhou University Second Hospital [approval number: 2023A‐733]. Informed consent was waived by the ethics committee due to the retrospective nature of the study. The research adhered to medical ethics standards.

### 2.2 Data Collection

Patient clinical data were collected from the electronic medical record system. Data collection were conducted by two independent researchers who reviewed patient charts and extracted key information, including demographic data, laboratory results, the TyG index, and mortality outcomes. Any discrepancies between the two reviewers were resolved through discussion and consensus, and all patient data were anonymized to ensure privacy protection. Baseline data included name, age, and gender. Vital signs recorded upon initial ICU admission included body temperature, heart rate, and others. Prior comorbidities included hypertension and whether multiple organ dysfunction syndrome (MODS) occurred within 48 h of ICU admission, among others. Initial serological indicators within 24 h of ICU admission included fasting glucose, fasting triglycerides, white blood cell count, and others. Ratio indicators comprised total cholesterol to high‐density lipoprotein (TC/HDL), low‐density lipoprotein to high‐density lipoprotein (LDL/HDL), triglyceride to high‐density lipoprotein (TG/HDL), and triglyceride to low‐density lipoprotein (TG/LDL). Scoring systems commonly used in sepsis management included the Acute Physiology and Chronic Health Evaluation II (APACHE II) and the Sequential Organ Failure Assessment (SOFA). Calculated indices consisted of the neutrophil‐to‐lymphocyte ratio (NLR), platelet‐to‐lymphocyte ratio (PLR), triglyceride‐glucose index (TyG), prognostic nutrition index (PNI), and systemic immune‐inflammation index (SII).

$$\mathrm{TyG}\;\text{index}=\ln\left[\frac{\text{fasting triglyceride}\;\left(\frac{\mathrm{mg}}{\mathrm{dl}}\right)\times \text{fasting blood glucose}\left(\frac{\mathrm{mg}}{\mathrm{dl}}\right)}{2}\right]$$

Prognostic nutritional indexPNI=serum albuming/L+5×total peripheral blood lymphocytes×10^9/L

$$\mathrm{SII}=\left(\text{Neutrophilcount}\times \text{Plateletcount}\right)/\text{Lymphocytecount}$$

Outcome: days in hospital, days in ICU, death in ICU, death in hospital, death within 28 days after ICU admission.

### 2.3 Statistical Analysis

Data were analyzed using SPSS 25.0 and R 4.4.1. Patients were randomly allocated into training and validation sets at a 7:3 ratio. In the training set, continuous variables were compared using the independent samples *t*‐test or Mann–Whitney *U* test based on distribution, while categorical variables were compared using the chi‐square test. Univariate and multivariate logistic regression analyses identified independent risk factors for 28‐day mortality. A predictive nomogram was subsequently constructed using R packages (mice, car, rms, pROC, devtools, DecisionCurve). Model performance was evaluated in both sets using receiver operating characteristic (ROC) curves, calibration curves, and decision curve analysis (DCA), with statistical significance set at *p* ≤ 0.05. For missing data, cases meeting exclusion criteria or lacking key variables were removed. Variables with > 30% missing data were excluded, while continuous variables with 5%–28% missing data were handled via multiple imputation.

## 3 Results

### 3.1 Patient Characteristics

A total of 578 patients were included in the study. The training cohort comprised 404 patients with a 28‐day mortality rate of 43.32%, whereas the validation cohort included 174 patients with a mortality rate of 40.80%. There were no significant differences in baseline characteristics between the two cohorts (all *p* > 0.05; Table 1).

**TABLE 1:** The clinical characteristics of the whole study population.

Variables	Total (*n* = 578)	Test (*n* = 174)	Train (*n* = 404)	*p*
Demographics				
Age	59.00 (49.00, 70.00)	59.00 (49.00, 71.00)	59.00 (49.00, 69.00)	0.558
Male, *n* (%)	379 (65.57)	114 (65.52)	265 (65.59)	0.986
BMI (Kg/m^2^)	22.86 (20.68, 25.38)	22.54 (20.25, 25.76)	22.86 (20.76, 24.87)	0.803
APACHEII	19.00 (15.00, 25.00)	19.00 (14.00, 26.00)	20.00 (15.00, 25.00)	0.721
SOFA	8.00 (6.00, 22.00)	9.00 (6.00, 22.00)	8.00 (6.00, 22.00)	0.234
Comorbidities				
Hypertension	151 (26.12)	51 (29.31)	100 (24.75)	0.253
Chronic lung disease	46 (7.96)	11 (6.32)	35 (8.66)	0.340
Coronary heart disease	32 (5.54)	9 (5.17)	23 (5.69)	0.802
Cerebrovascular disease	32 (5.54)	12 (6.90)	20 (4.95)	0.348
Organ dysfunction				
Respiratory failure in 48 h	354 (61.25)	102 (58.62)	252 (62.38)	0.395
Heart Failure in 48 h	135 (23.36)	44 (25.29)	91 (22.52)	0.471
Renal Failure in 48 h	270 (46.71)	89 (51.15)	181 (44.80)	0.161
MODS in 48 h	254 (43.94)	79 (45.40)	175 (43.32)	0.643
Medical measurements				
HR (bpm)	108.00 (94.00, 126.00)	107.50 (89.00, 123.75)	108.00 (95.00, 128.00)	0.166
MAP (mmHg)	77.50 (64.08, 92.67)	78.83 (67.75, 93.50)	76.50 (62.58, 92.00)	0.066
RR (rpm)	24.00 (20.00, 31.00)	25.00 (20.00, 31.00)	24.00 (20.00, 31.00)	0.929
SPO2	93.00 (88.00, 96.00)	93.00 (89.00, 97.00)	92.50 (87.00, 96.00)	0.206
Temperature (°C)	36.90 (36.50, 37.70)	36.80 (36.50, 37.60)	37.00 (36.50, 37.80)	0.431
Laboratory tests				
WBC (×10^9^/L)	11.76 (6.92, 18.10)	11.06 (6.80, 18.62)	11.98 (6.95, 17.83)	0.950
NE%	0.90 (0.84, 0.93)	0.89 (0.84, 0.93)	0.90 (0.84, 0.93)	0.703
Hb (g/L)	120.00 (99.00, 143.00)	121.50 (97.25, 140.75)	120.00 (99.00, 144.00)	0.709
PLT (×109/L)	114.00 (60.25, 184.00)	108.00 (56.50, 180.50)	122.00 (61.00, 184.50)	0.307
HCT	0.36 (0.30, 0.43)	0.37 (0.29, 0.42)	0.36 (0.30, 0.43)	0.502
RDW‐SD (%)	47.00 (44.00, 52.00)	47.00 (44.00, 50.75)	47.00 (44.00, 52.00)	0.196
RDW‐CV (%)	13.80 (13.10, 15.10)	13.70 (12.90, 14.90)	13.80 (13.20, 15.10)	0.214
CRP	130.40 (61.20, 197.19)	127.59 (55.03, 179.35)	132.06 (63.09, 207.46)	0.311
Serum uric acid (μmol/L)	323.50 (213.00, 440.75)	313.75 (218.00, 451.62)	328.00 (212.00, 439.00)	0.694
Urea nitrogen (mmol/L)	10.80 (7.30, 16.69)	10.50 (7.25, 16.70)	10.89 (7.36, 16.54)	0.686
Creatinine (μmol/L)	106.45 (69.17, 184.02)	105.95 (69.32, 199.15)	106.60 (69.25, 177.78)	0.751
TB (μmol/L)	25.15 (14.75, 45.27)	23.95 (14.53, 46.35)	25.35 (14.97, 44.73)	0.692
DB (μmol/L)	11.60 (5.73, 27.08)	11.20 (5.30, 24.50)	11.70 (5.90, 27.23)	0.851
ALT (U/L)	38.00 (21.00, 83.00)	35.50 (20.25, 80.75)	38.00 (21.00, 83.50)	0.440
AST (U/L)	53.00 (31.00, 119.75)	50.00 (31.25, 108.75)	56.50 (30.00, 130.25)	0.492
ALB (g/L)	28.37 ± 5.04	28.53 ± 5.17	28.30 ± 4.99	0.622
LDL (mmol/L)	1.09 (0.67, 1.59)	1.07 (0.68, 1.46)	1.09 (0.66, 1.70)	0.489
HDL (mmol/L)	0.57 (0.38, 0.84)	0.59 (0.39, 0.86)	0.56 (0.37, 0.83)	0.550
PCT (ng/ml)	15.95 (2.66, 63.36)	11.79 (2.40, 40.49)	18.60 (2.76, 70.82)	0.064
Dimer (umol/L)	7.02 (2.80, 17.45)	7.45 (2.76, 17.35)	6.89 (2.83, 17.37)	0.980
INR	1.35 (1.15, 1.56)	1.33 (1.13, 1.56)	1.35 (1.16, 1.56)	0.574
FIB (g/L)	4.10 (2.80, 5.40)	3.88 (2.69, 5.29)	4.24 (2.99, 5.41)	0.076
Lac (mmol/L)	2.50 (1.40, 4.80)	2.15 (1.40, 4.20)	2.50 (1.50, 5.32)	0.167
NLR	16.36 (7.98, 27.68)	16.17 (7.85, 26.24)	16.76 (8.31, 29.42)	0.412
TyG	8.59 (8.16, 9.06)	8.58 (8.15, 9.11)	8.59 (8.16, 9.00)	0.624
PLR	181.70 (89.32, 341.32)	174.44 (81.40, 327.78)	192.11 (96.76, 355.23)	0.253
SII	1633.87 (692.92, 3532.27)	1479.68 (706.36, 3120.55)	1720.31 (690.85, 3620.50)	0.287
PNI	31.75 (28.20, 35.50)	31.77 (28.75, 36.15)	31.75 (28.15, 35.20)	0.279
TC/HDL	3.43 (2.48, 5.03)	3.37 (2.42, 4.97)	3.45 (2.50, 5.04)	0.320
LDL/HDL	1.89 (1.13, 3.19)	1.77 (1.14, 2.70)	1.91 (1.12, 3.32)	0.218
TG/HDL	1.90 (1.03, 4.15)	1.82 (1.03, 4.37)	1.95 (1.03, 4.05)	0.770
TG/LDL	0.93 (0.65, 1.70)	0.93 (0.64, 2.06)	0.93 (0.65, 1.63)	0.666
Outcomes				
In‐hospital mortality	100 (17.30)	33 (18.97)	67 (16.58)	0.488
ICU mortality	98 (16.96)	33 (18.97)	65 (16.09)	0.398
28‐day mortality	246 (42.56)	71 (40.80)	175 (43.32)	0.575
Hospital‐los‐day	12.00 (6.00, 21.00)	12.50 (6.00, 24.00)	11.50 (6.00, 21.00)	0.185

### 3.2 Univariate and Multivariate Regression Analysis in the Training Set

Variables significantly associated with 28‐day mortality in univariate regression analysis (*p* < 0.05) were included in a multivariate logistic regression model. The results identified the following six independent predictors of 28‐day mortality in sepsis patients: age, respiratory failure within 48 h, multiple organ dysfunction syndrome within 48 h, lactate level, international normalized ratio (INR), and the triglyceride‐glucose (TyG) index. These variables were incorporated into the final predictive model and subjected to correlation analysis. While the Prognostic Nutritional Index (PNI) was significant in the univariate analysis, it did not retain independent significance in the multivariate analysis, which may be attributed to the limited sample size. Detailed results are presented in Table 2.

**TABLE 2:** Results of univariate logistic regression and multifactorial logistic regression analysis of training sets.

Variables	Univariate logistic regression analysis					Multifactorial logistic regression analysis				
	β	SE	*Z*	*p*	OR (95% CI)	β	SE	*Z*	*p*	OR (95% CI)
Age	0.02	0.01	3.21	0.001*	1.02 (1.01~1.03)	0.04	0.01	3.65	< 0.001*	1.04 (1.02~1.06)
Male, *n* (%)	0.01	0.21	0.04	0.965	1.01 (0.67~1.53)	—	—	—	—	—
BMI(Kg/m^2^)	−0.01	0.03	−0.43	0.664	0.99 (0.94~1.04)	—	—	—	—	—
Hypertension	0.36	0.23	1.55	0.121	1.43 (0.91~2.25)	—	—	—	—	—
Chronic lung disease	0.61	0.36	1.71	0.088	1.84 (0.91~3.71)	—	—	—	—	—
Coronary heart disease	0.38	0.43	0.88	0.380	1.46 (0.63~3.39)	—	—	—	—	—
Cerebrovascular disease	0.93	0.48	1.95	0.052	2.54 (0.99~6.52)	—	—	—	—	—
Respiratory failure in 48 h	1.54	0.23	6.57	< 0.001*	4.66 (2.94~7.37)	1.15	0.31	3.78	< 0.001*	3.17 (1.74~5.77)
Heart failure in 48 h	1.08	0.25	4.37	< 0.001*	2.94 (1.81~4.78)	0.40	0.33	1.21	0.225	1.48 (0.78~2.81)
Renal failure in 48 h	1.15	0.21	5.49	< 0.001*	3.15 (2.09~4.75)	0.20	0.34	0.60	0.546	1.23 (0.63~2.38)
MODS in 48 h	1.88	0.22	8.42	< 0.001*	6.56 (4.24~10.17)	1.15	0.33	3.46	< 0.001*	3.15 (1.64~6.05)
HR	0.01	0.00	3.17	0.002*	1.01 (1.01~1.02)	0.01	0.01	1.13	0.257	1.01 (1.00~1.02)
MAP	−0.00	0.00	−1.05	0.292	1.00 (0.99~1.00)	—	—	—	—	—
RR	0.01	0.01	0.88	0.379	1.01 (0.99~1.03)	—	—	—	—	—
SPO2	−0.07	0.01	−4.91	< 0.001*	0.94 (0.91~0.96)	−0.02	0.02	−1.20	0.231	0.98 (0.95~1.01)
Temperature	−0.03	0.10	−0.32	0.748	0.97 (0.79~1.18)	—	—	—	—	—
WBC	0.02	0.01	1.42	0.156	1.02 (0.99~1.04)	—	—	—	—	—
Ne%	−0.14	0.81	−0.17	0.864	0.87 (0.18~4.22)	—	—	—	—	—
Hb	−0.00	0.00	−0.63	0.529	1.00 (0.99~1.00)	—	—	—	—	—
PLT										
> 300	—	—	—	—	1.00 (reference)	—	—	—	—	—
100–300	0.42	0.40	1.06	0.289	1.52 (0.70~3.31)	—	—	—	—	—
< 100	0.51	0.40	1.27	0.205	1.66 (0.76~3.63)	—	—	—	—	—
HCT	−0.06	1.06	−0.06	0.956	0.94 (0.12~7.53)	—	—	—	—	—
RDW‐SD	−0.00	0.00	−0.50	0.619	1.00 (0.99~1.01)	—	—	—	—	—
RDW‐CV	0.03	0.04	0.96	0.335	1.03 (0.97~1.11)	—	—	—	—	—
CRP (mg/L)										
< 131	—	—	—	—	1.00 (reference)	—	—	—	—	1.00 (reference)
≥ 131	0.41	0.20	2.04	0.041*	1.51 (1.02~2.25)	0.37	0.27	1.37	0.171	1.45 (0.85~2.46)
Serum uric acid										
< 323	—	—	—	—	1.00 (reference)	—	—	—	—	—
≥ 323	0.40	0.20	1.96	0.050	1.49 (1.00~2.21)	—	—	—	—	—
Urea nitrogen	0.03	0.01	2.40	0.017*	1.03 (1.01~1.05)	0.02	0.01	1.57	0.116	1.02 (1.00~1.04)
Creatinine(μmol/L)										
< 133	—	—	—	—	1.00 (Reference)	—	—	—	—	1.00 (reference)
≥ 133	0.65	0.21	3.09	0.002*	1.91 (1.27~2.88)	−0.57	0.34	−1.69	0.091	0.57 (0.29~1.10)
TB	0.00	0.00	1.21	0.227	1.00 (1.00~1.01)	—	—	—	—	—
DB	0.00	0.00	1.90	0.058	1.00 (1.00~1.01)	—	—	—	—	—
ALT										
< 38	—	—	—	—	1.00 (reference)	—	—	—	—	—
≥ 38	0.40	0.20	1.96	0.050	1.49 (1.00~2.21)	—	—	—	—	—
AST										
< 53	—	—	—	—	1.00 (reference)	—	—	—	—	1.00 (reference)
≥ 53	0.69	0.20	3.38	< 0.001*	1.99 (1.34~2.98)	0.19	0.28	0.67	0.501	1.21 (0.70~2.08)
ALB	−0.12	0.02	−5.30	< 0.001*	0.89 (0.85~0.93)	−0.04	0.05	−0.75	0.453	0.96 (0.87~1.06)
TC	0.02	0.07	0.27	0.786	1.02 (0.89~1.17)	—	—	—	—	—
LDL	−0.17	0.13	−1.33	0.183	0.84 (0.66~1.08)	—	—	—	—	—
HDL	−0.45	0.28	−1.60	0.109	0.64 (0.37~1.10)	—	—	—	—	—
PCT	−0.00	0.00	−1.45	0.146	1.00 (0.99~1.00)	—	—	—	—	—
Dimer	0.00	0.00	0.51	0.608	1.00 (1.00~1.01)	—	—	—	—	—
INR	0.91	0.25	3.70	< 0.001*	2.49 (1.54~4.05)	0.67	0.34	2.01	0.045*	1.96 (1.02~3.78)
FIB	−0.06	0.05	−1.22	0.224	0.94 (0.85~1.04)	—	—	—	—	—
LAC	0.19	0.04	5.37	< 0.001*	1.21 (1.13~1.30)	0.12	0.05	2.59	0.010*	1.13 (1.03~1.24)
NLR	0.00	0.00	0.58	0.565	1.00 (0.99~1.01)	—	—	—	—	—
PLR										
< 182	—	—	—	—	1.00 (reference)	—	—	—	—	—
≥ 182	−0.16	0.20	−0.80	0.426	0.85 (0.57~1.26)	—	—	—	—	—
SII										
< 1634	—	—	—	—	1.00 (reference)	—	—	—	—	—
≥ 1634	0.04	0.20	0.18	0.856	1.04 (0.70~1.54)	—	—	—	—	—
PNI	−0.09	0.02	−4.63	< 0.001*	0.92 (0.88~0.95)	−0.06	0.04	−1.48	0.140	0.94 (0.87~1.02)
TyG	0.84	0.17	4.99	< 0.001*	2.31 (1.66~3.22)	0.97	0.24	4.05	< 0.001*	2.65 (1.65~4.24)

*Note:* * denotes *p* < 0.05, indicating a statistically significant association in the regression analysis.

Abbreviations: CI: confidence interval; SE: standard error; *Z*: *Z*‐score; *p*: *p*‐value OR: odds ratio; β: Beta, regression coefficient.

### 3.3 Development and Comparison of Prediction Models

Model 1 was constructed based on the six predictors identified by multivariable logistic regression. Given the significant association of the PNI with prognosis in univariate analysis and its established utility in predicting infections in the elderly, it was incorporated to develop Model 2. To specifically evaluate the TyG index, Model 3 was built containing this marker alone. For comparison, Models 4 and 5 were developed using only the APACHE II score and SOFA score, respectively. All variables in each model showed statistical significance (*p* < 0.05; Table S1). ROC analysis (Figure 1A) revealed that the area under the curve (AUC) values for the five models in the training set were 0.842, 0.855, 0.671, 0.729, and 0.660, respectively (Table 3). Compared to traditional ICU severity scores (APACHE II and SOFA), both Model 1 and Model 2 demonstrated superior predictive performance with a more streamlined variable set. Between these two, Model 2 exhibited a slightly higher AUC. Considering its enhanced predictive value and the clinical practicality of readily obtaining PNI, Model 2 was selected for the subsequent construction of the clinical nomogram.

**FIGURE 1:**
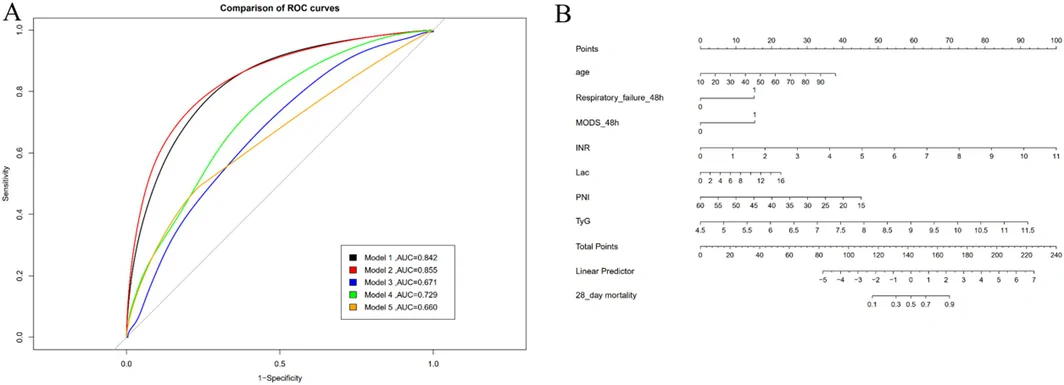
ROC curves of five predictive models and nomogram for 28‐day mortality risk in sepsis patients. (A) Comparison of ROC curves in training set. Model 1: Age, respiratory failure within 48 h, multiple organ dysfunction syndrome within 48 h, lactate, INR, and TyG index. Model 2: All variables in Model 1 plus the PNI. Model 3: TyG index alone. Model 4: APACHE II score. Model 5: SOFA score. (B) Construction of a nomogram for predicting 28‐day mortality in patients with sepsis (Respiratory_failure_48h 0 = Not Occurred, 1 = Occurred; MODS_48h 0 = Not Occurred, 1 = Occurred).

**TABLE 3:** The confusion matrix of every model in the training set.

Model	AUC (95% CI)	Accuracy (95% CI)	Sensitivity (95% CI)	Specificity (95% CI)	PPV (95% CI)	NPV (95% CI)	Cut off
1	0.842 (0.803–0.880)	0.770 (0.726–0.810)	0.769 (0.714–0.823)	0.771 (0.709–0.834)	0.815 (0.763–0.867)	0.718 (0.654–0.782)	0.429
2	0.855 (0.818–0.891)	0.787 (0.744–0.826)	0.834 (0.786–0.882)	0.726 (0.660–0.792)	0.799 (0.748–0.850)	0.770 (0.705–0.834)	0.505
3	0.671 (0.619–0.724)	0.634 (0.585–0.681)	0.590 (0.526–0.653)	0.691 (0.623–0.760)	0.714 (0.650–0.779)	0.563 (0.496–0.629)	8.535
4	0.729 (0.680–0.777)	0.676 (0.628–0.721)	0.646 (0.584–0.708)	0.714 (0.647–0.781)	0.747 (0.687–0.808)	0.607 (0.540–0.673)	19.5
5	0.660 (0.606–0.714)	0.666 (0.618–0.712)	0.843 (0.796–0.890)	0.434 (0.361–0.508)	0.661 (0.607–0.715)	0.679 (0.592–0.765)	21

*Note:* Performance metrics include the area under the curve (AUC), accuracy, sensitivity, specificity, positive predictive value (PPV), negative predictive value (NPV) (all with 95% confidence intervals), and the optimal cutoff value.

In addition, Table S2 shows that adding the TyG index to APACHE II or SOFA scores improves model performance (AUC, NRI, IDI). Yet, these combined models' AUCs (0.761 and 0.695) are still lower than that of the comprehensive Model 2 (AUC = 0.855), highlighting the value of multivariable prediction.

### 3.4 Establishment and Internal Validation of the Nomogram

A prognostic nomogram (Figure 1B) was constructed using R software. The variables included, from top to bottom, are age, respiratory failure within 48 h, multiple organ dysfunction syndrome within 48 h, lactate level, INR, TyG index, PNI, linear predictor, and 28‐day mortality. Each variable corresponds to a calibrated axis where specific values are assigned scale points. The total score, obtained by summing the points from all variables, predicts the individual's 28‐day mortality risk. According to the nomogram, a total score of 140 points corresponds to an approximate 50% probability of 28‐day mortality in sepsis patients.

The regression model was constructed according to the factors included in the nomogram, and the ROC curve was drawn using the data of the training set and the validation set. The AUC values in the two sets of data were 0.855 in the training set and 0.826 in the validation set(Figure 2).

**FIGURE 2:**
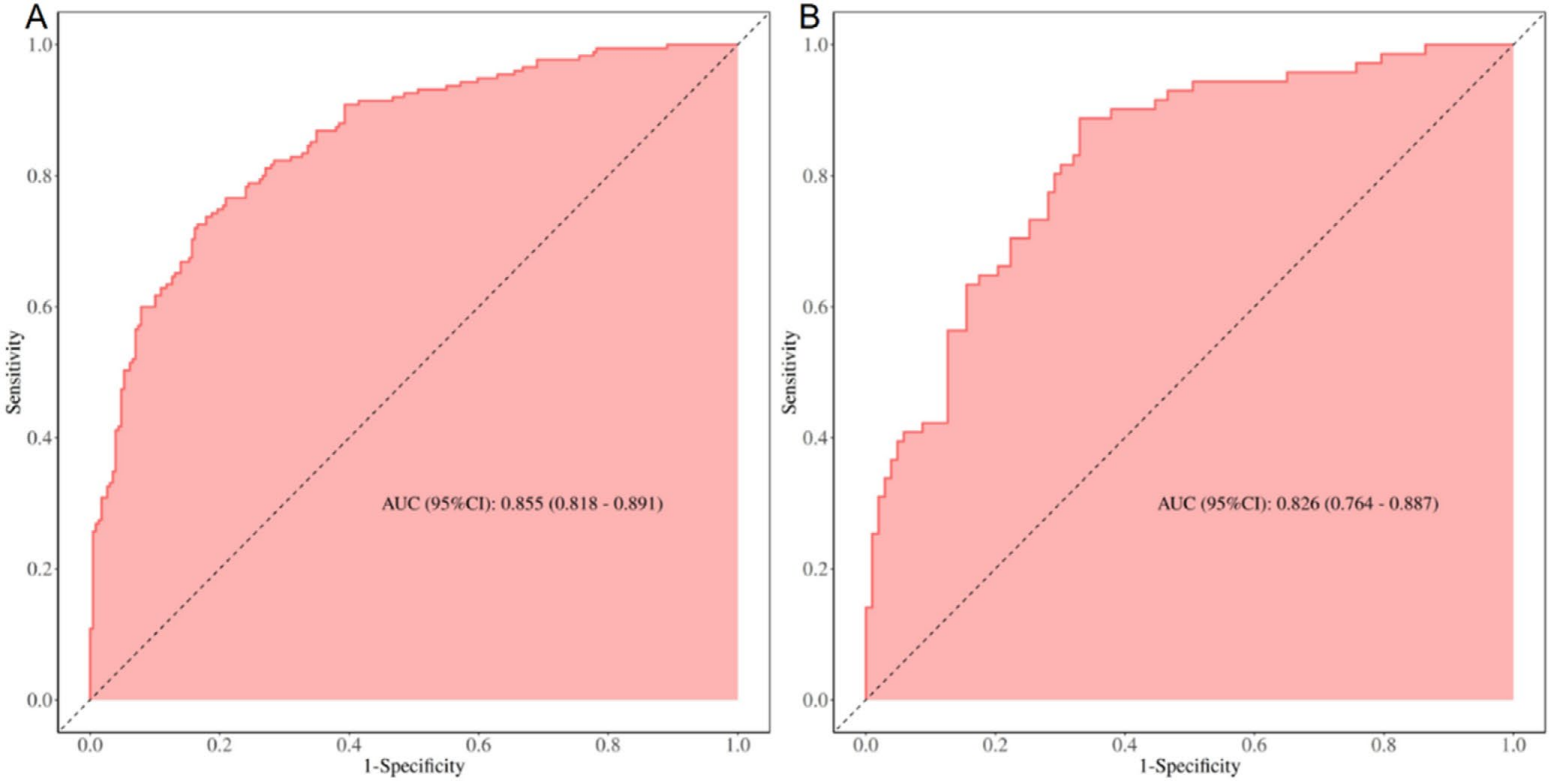
Comparison of ROC curves for the predictive model in the training and validation sets. (A) ROC curves of the nomogram in the training cohort. (B) ROC curves of the nomogram in the validation cohort.

Calibration curves were plotted using data from both the training and validation sets. The figures show good agreement between the actual and predicted curves for both datasets, indicating reliable prediction probability (Figure 3A,B). DCA was performed to assess the clinical utility of the model and its impact on practical decision‐making. The results from both the training and validation sets demonstrated that the predictive model offers significant clinical benefit (Figure 3C,D).

**FIGURE 3:**
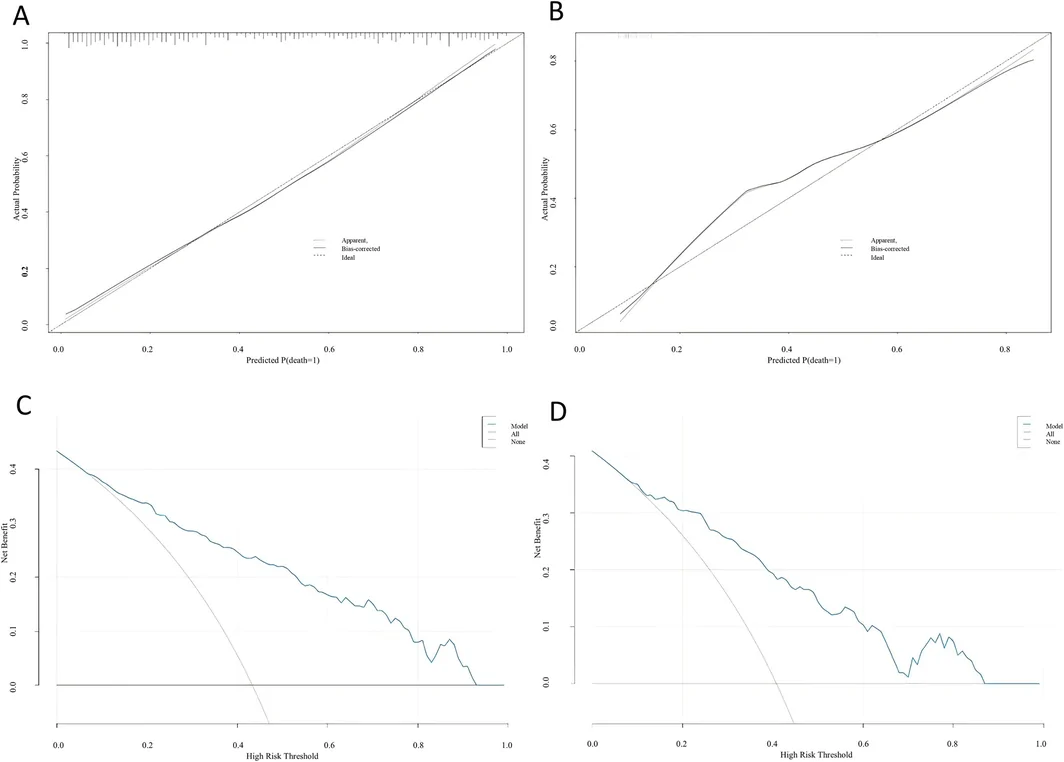
Calibration and decision curve analysis. (A) Calibration curve of the nomogram in the training cohort. (B) Calibration curve of the nomogram in the validation cohort. (C) DCA in the training cohort. (D) DCA in the validation cohort.

## 4 Discussion

Sepsis remains a leading cause of mortality in critically ill patients, underscoring the critical importance of early and accurate assessment of disease severity and prognosis for timely clinical intervention [1, 2]. Conventional scoring systems exhibit limitations in evaluating glucose metabolism disorders and insulin resistance. It is well established that heightened insulin resistance is associated with increased short‐term mortality risk in sepsis patients [9]. In this study, we were the first to demonstrate in a Western China sepsis cohort that an elevated TyG index is significantly associated with higher 28‐day mortality. Accordingly, we developed a prognostic nomogram incorporating seven clinical variables—age, respiratory failure within 48 h, multiple organ dysfunction syndrome within 48 h, lactate level, INR, TyG index, and PNI—to predict 28‐day mortality. This model demonstrated superior predictive performance compared to conventional scoring systems and relies on readily available clinical parameters. The inclusion of the TyG index, a key early indicator of insulin resistance, carries significant clinical relevance [9, 10].

The TyG index has emerged as a significant biomarker associated with various disease risks, including metabolic disorders [7], myocardial infarction [11], and ischemic stroke [12]. Clinical evidence consistently links elevated TyG index levels with increased mortality in critically ill populations. For instance, studies by Zhang and Park identify the TyG index as a reliable biomarker for severe pancreatitis [13, 14]. Liao reported a significant association between the TyG index and all‐cause mortality in critically ill patients [15]. Furthermore, Zheng's research indicates that in patients with sepsis, each unit increase in the TyG index is associated with a 44% increase in the risk of in‐hospital mortality [9]. Corroborating these findings, our analysis in a Western China population confirms the TyG index as an independent predictor for 28‐day mortality in septic patients, with a predictive value of 0.671 (95% CI: 0.619–0.724). In a Western ICU cohort derived from the MIMIC‐IV database (*n* = 1257), a higher TyG index was independently associated with 28‐day mortality (OR 1.391, 95% CI 1.152–1.678), with a linear increase in mortality risk observed across increasing TyG levels [9]. Similar associations were observed in the present study.

The TyG index is closely linked to insulin resistance (IR), a well‐established contributor to endothelial dysfunction, oxidative stress, immune dysregulation, coagulation abnormalities, and enhanced inflammatory responses [16, 17]. The degree of systemic inflammation, closely correlated with IR, is a critical determinant of sepsis prognosis. Sepsis itself can induce IR and disturb lipid metabolism, often manifesting as uncontrolled hyperglycemia and significant glycemic variability during the acute phase [18]. Previous studies have suggested that the TyG index is associated with systemic inflammation. In hospitalized patients, TyG has been positively correlated with CRP and TNFα; in critically ill patients with sepsis, inflammatory markers tend to increase at higher TyG levels [19, 20]. Our study similarly observed a significant upward trend in C‐reactive protein and white blood cell counts with increasing TyG index. Although the precise biological mechanisms linking the TyG index to sepsis outcomes require further elucidation, it is hypothesized that acute‐phase alterations in IR may reflect the underlying inflammatory state and disease severity [9]. Therefore, the TyG index is recognized in this study as a key prognostic indicator, potentially offering valuable insights for the risk assessment and management of sepsis.

The nomogram constructed in this study incorporates multiple predictors with clear clinical and pathophysiological relevance. PNI, a composite biomarker reflecting nutritional status and systemic inflammation, has been validated as an effective indicator for predicting both short‐term and long‐term outcomes in sepsis patients. Moreover, this parameter demonstrated statistical significance in the univariate analysis, and its inclusion in the model enhances its comprehensive assessment capability. Therefore, it was incorporated into the prognostic prediction model in this study [21, 22, 23, 24]. Furthermore, age is an independent risk factor for prognosis [24]. Respiratory failure and MODS occurring within 48 h directly reflect disease severity and organ injury [25, 26]. The INR indicates coagulopathy and the risk of disseminated intravascular coagulation [27], while lactate level serves as a key metabolic marker for assessing tissue perfusion and cellular hypoxia [28, 29]. Together, these parameters provide a comprehensive and readily accessible clinical basis for early risk stratification and prognostic evaluation in sepsis patients from multiple dimensions, including inflammation, metabolism, organ function, coagulation, and perfusion.

### 4.1 Strengths and Limitations

This study is the first to validate the TyG index for predicting 28‐day mortality in a Western China cohort. The developed nomogram, which utilizes readily available clinical indicators, demonstrates good applicability and facilitates individualized risk stratification, offering a practical tool for clinical decision‐making. However, several limitations should be acknowledged. First, the single‐center nature of this study may limit the generalizability of our findings, highlighting the need for validation in multicenter cohorts. Second, the retrospective design is inherently susceptible to selection bias. Moreover, our model did not incorporate certain critical therapeutic factors (e.g., antimicrobial therapy) or time‐dependent covariates, which may influence outcome prediction. Furthermore, the observed mortality rate in this study should be interpreted in the specific context of our cohort: as a regional referral center, our ICU often admits patients with advanced organ dysfunction, likely contributing to higher baseline severity. Additionally, although the mean age was 59 years, 39.4% of patients were aged ≥ 65 years, a subgroup with typically higher comorbidity burden and mortality risk. Therefore, caution is warranted when extrapolating these findings to other settings. Future multicenter, prospective studies are warranted to further validate the generalizability and clinical applicability of this model. In addition, exploration of the dynamic changes of the TyG index during sepsis progression may provide further mechanistic insight.

## 5 Conclusion

This study confirms that the TyG index serves as a predictor of 28‐day mortality risk in sepsis patients. Based on this finding, we developed and validated a predictive model for early identification of high‐risk individuals. The variables included in the model are readily obtainable in clinical practice, facilitating timely intervention and providing a practical tool to improve patient outcomes across diverse healthcare settings. Furthermore, exploring potential therapeutic strategies targeting sepsis‐associated insulin resistance may open new avenues for optimizing current treatment regimens and enhancing patient prognosis.

## Funding

This study was supported by the National Natural Science Foundation of China (82560378) and the Gansu Provincial Department of Science and Technology (Gansu Province Joint Scientific Research Fund) (24JRRA930).

## Conflicts of Interest

The authors declare no conflicts of interest.

## Supporting information


**Figure S1:** Study flowchart: inclusion, grouping, and analysis.

**Table S1:** Assessing the significance of each component in models.
**Table S2:** Incremental value of the TyG index added to conventional severity scores.

## Data Availability

The data that support the findings of this study are available on request from the corresponding author. The data are not publicly available due to privacy or ethical restrictions.
