# Supplementary figures and images for: Triglyceride‐Glucose Index‐Based Nomogram for Predicting Short‐Term Mortality in Sepsis Patients
Source: Kaohsiung J Med Sci. 2026 Apr 2:e70199. Online ahead of print. doi: 10.1002/kjm2.70199 (PMC13399648; doi:10.1002/kjm2.70199)

**Supplementary Figure 1.** **Study Flowchart: Inclusion, Grouping, and Analysis**


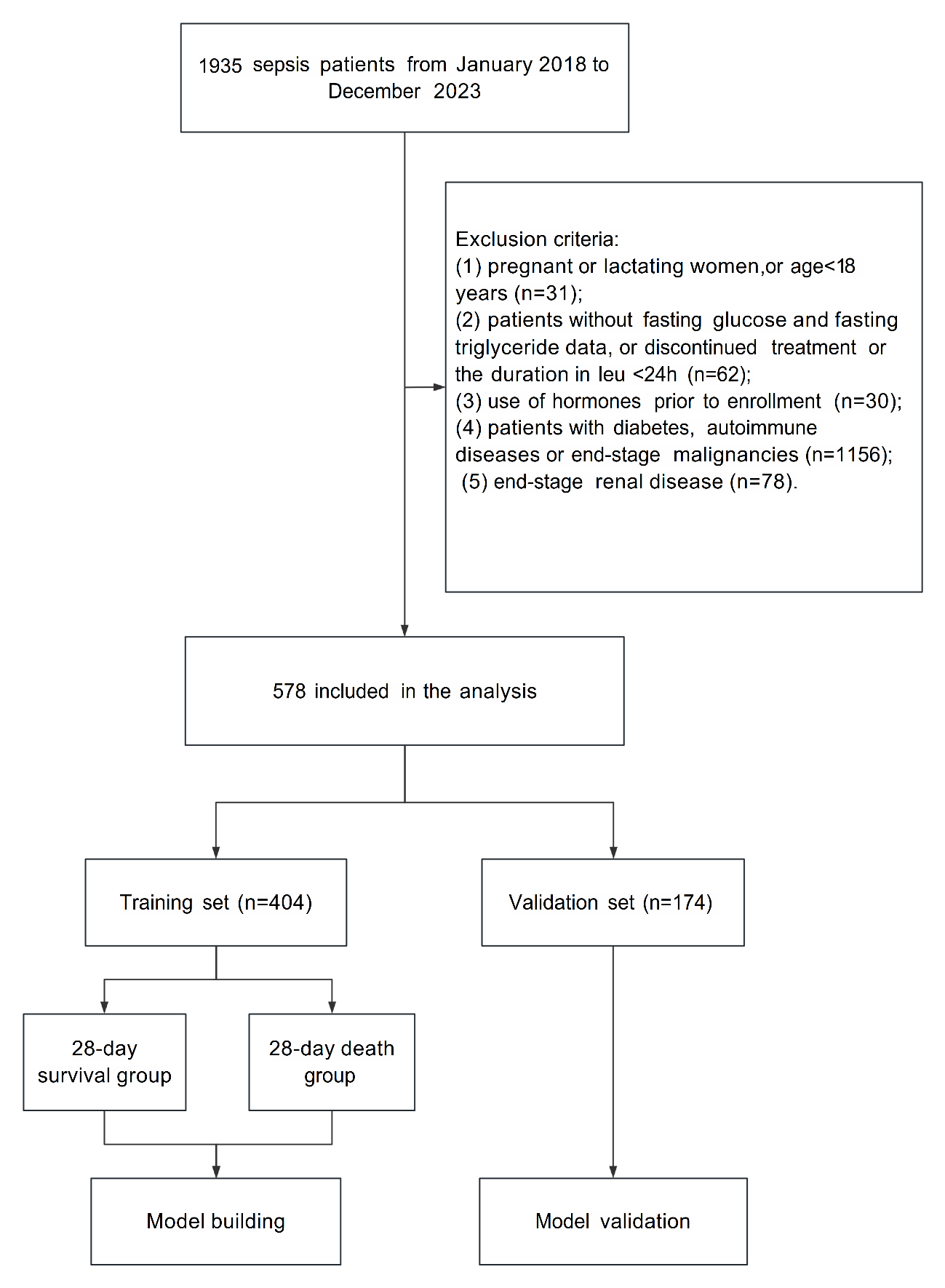

Supplement: Supplementary file 1 — Figure S1: Study flowchart: inclusion, grouping, and analysis. [file KJM2-9999-e70199-s002.docx]
