# Supplementary material for: Triglyceride‐Glucose Index‐Based Nomogram for Predicting Short‐Term Mortality in Sepsis Patients
Source: Kaohsiung J Med Sci. 2026 Apr 2:e70199. Online ahead of print. doi: 10.1002/kjm2.70199 (PMC13399648; doi:10.1002/kjm2.70199)
Supplement: Supplementary file 2 — Table S1: Assessing the significance of each component in models. Table S2: Incremental value of the TyG index added to conventional severity scores. [file KJM2-9999-e70199-s001.docx]

**Supplementary Table 1. Assessing the significance of each component in Models**

|  |  | OR | 95% | P |
| --- | --- | --- | --- | --- |
| Model 1 | Age | 1.03 | 1.02 ~ 1.05 | <0.001 |
|  | Respiratory failure in 48h | 3.51 | 2.03 ~ 6.08 | <0.001 |
|  | MODS in 48h | 3.64 | 2.17 ~ 6.11 | <0.001 |
|  | Lac | 1.14 | 1.05 ~ 1.23 | 0.003 |
|  | INR | 2.41 | 1.23 ~ 4.71 | 0.010 |
|  | TyG | 2.89 | 1.91 ~ 4.36 | <0.001 |
|  |  | OR | 95% | P |
| Model 2 | Age | 1.04 | 1.02 ~ 1.06 | <0.001 |
|  | Respiratory failure in 48h | 3.59 | 2.05 ~ 6.28 | <0.001 |
|  | MODS in 48h | 3.62 | 2.13 ~ 6.16 | <0.001 |
|  | Lac | 1.13 | 1.03 ~ 1.23 | 0.008 |
|  | INR | 2.15 | 1.10 ~ 4.20 | 0.025 |
|  | TyG | 3.03 | 1.98 ~ 4.62 | <0.001 |
|  | PNI | 0.92 | 0.88 ~ 0.96 | <0.001 |
|  |  | OR | 95% | P |
| Model 3 | TyG | 2.31 | 1.66 ~ 3.22 | <0.001 |
|  |  | OR | 95% | P |
| Model 4 | APACHEII | 1.13 | 1.09 ~ 1.17 | <0.001 |
|  |  | OR | 95% | P |
| Model 5 | SOFA | 1.08 | 1.05 ~ 1.10 | <0.001 |

Note: Model 1: Age +Respiratory failure in 48h +Lac + MODS in 48h +INR +TyG; Model 2: Age +Respiratory failure in 48h +Lac + MODS in 48h +INR +Ty G +PNI; Model 3: TyG; Model 4: APACHEII; Model 5: SOFA

**Supplementary Table 2. Incremental value of the TyG index added to conventional severity scores.**

| **Model** | **AUC (95% CI)** | **ΔAUC (P value)** | **NRI (95% CI)** | **IDI (95% CI)** |
| --- | --- | --- | --- | --- |
| APACHII | 0.729 (0.680-0.777) | Reference | — | — |
| APACHII + TyG | **0.761 (0.718–0.810)** | +0.032 (P < 0.001) | 0.437 (0.242–0.627) | 0.043 (0.024–0.062) |
| SOFA | 0.660 (0.606-0.714) | Reference | — | — |
| SOFA + TyG | 0.695 (0.658-0.755) | +0.035 (P < 0.001) | 0.460 (0.258–0.648) | 0.048 (0.028–0.069) |

The addition of the TyG index to either APACHE II or SOFA scores significantly improved model discrimination and reclassification, as shown by the increase in the area under the curve (AUC), net reclassification improvement (NRI), and integrated discrimination improvement (IDI). Data are presented as AUC, NRI, and IDI with their 95% confidence intervals (CI). ΔAUC represents the change in AUC, with the corresponding P value from DeLong's test.
